# FAMILY CAREGIVING AND THE OLDER AMERICANS ACT

**DOI:** 10.1093/geroni/igz038.856

**Published:** 2019-11-08

**Authors:** C Grace Whiting

**Affiliations:** National Alliance for Caregiving, Bethesda, Maryland, United States

## Abstract

This session will focus on the challenges faced by millions of family caregivers and the innovative approaches that the OAA and other programs are using to be supportive of caregivers.

